# Determinants of Parental Adherence to Childhood Immunization Among Children Under Five in Marginalized Asian Populations

**DOI:** 10.3390/ijerph22111692

**Published:** 2025-11-09

**Authors:** Nitima Nulong, Nirachon Chutipattana, Lan Thi Kieu Nguyen, An Dai Tran, Uyen Thi To Nguyen, Cua Ngoc Le

**Affiliations:** 1 Excellent Center for Public Health Research, School of Public Health, Walailak University, 222 Thaiburi, Nakhon Sithammarat 80160, Thailand; n.nitima@gmail.com (N.N.); nirachon.ch@wu.ac.th (N.C.); 2 Master in Public Health Research Program, School of Public Health, Walailak University, 222 Thaiburi, Nakhon Sithammarat 80160, Thailand; ntklan@ctump.edu.vn; 3 Dong Thap Provincial Center for Disease Control, Dong Thap 8100, Vietnam; daiandt2016@gmail.com; 4 Dong Thap Medical College, Dong Thap 8100, Vietnam; nttuyen@cdytdt.edu.vn

**Keywords:** adherence, childhood immunization, marginalized populations, Asia

## Abstract

Childhood immunization is one of the most effective public health measures, yet inequities remain in marginalized populations across Asia, where parental adherence is essential to sustaining the Expanded Program on Immunization. This narrative review examines determinants of adherence among under-five children in disadvantaged communities. Following PRISMA guidelines, searches of PubMed, Scopus, and Google Scholar identified studies published between 2015 and 2025, with earlier key works included as relevant. Twenty-one studies from South, Southeast, and East Asia were analyzed. Five domains were associated with adherence: socioeconomic and access factors, where maternal education, household income, and possession of immunization cards were positive predictors, while remote residence was a barrier; trust, cultural beliefs, and social norms, with misinformation and vaccine controversies reducing uptake, and provider trust and supportive norms improving it; migration and mobility, as migrant, stateless, and left-behind children had lower coverage due to weak registration and disrupted caregiving; household and caregiver dynamics, where decision-making by family or community members shaped uptake, while large family size and maternal employment limited adherence; and health system capacity, with inadequate infrastructure and follow-up hindering coverage and integration with maternal–child health services facilitating it. Addressing these intersecting barriers through equity-focused strategies is critical to achieving universal immunization coverage.

## 1. Introduction

Childhood immunization is one of the most effective public health interventions for reducing morbidity and mortality from vaccine-preventable diseases (VPDs) [1]. Despite global efforts through programs like the World Health Organization’s Expanded Program on Immunization (EPI), disparities in vaccine uptake persist [2]—particularly among marginalized populations in Asia. These populations often live in geographically remote, socioeconomically disadvantaged, or politically marginalized contexts, compounding barriers to immunization access and adherence [3]. Marginalized groups in Asia include slum communities under overcrowding, informal housing, poor sanitation, and limited public services.Haor communities in the wetland areas of Bangladesh include residents who often disrupt healthcare access in flooding seasons.Rohingya refugees refer to a stateless Muslim minority displaced from Myanmar and living in often-overcrowded settlements.Makeshift camps refer to temporary, unregistered refugee shelters that lack formal infrastructure.And left-behind children refer to those whose parents have migrated for work—often across borders or to urban areas, leaving children in the care of relatives or guardians, with inconsistent health service follow-up.

Parental adherence to immunization schedules plays a pivotal role in ensuring timely protection of children under five years of age. However, parental decisions are shaped by a complex interplay of factors that include cultural beliefs, trust in health systems, perceived vaccine efficacy, and experiences with health service delivery. In ethnic minority communities, traditional norms, language barriers, and historical exclusion from mainstream health campaigns may further hinder vaccine acceptance and completion [4].

Asia has a diverse array of marginal groups and offers a unique landscape for exploring how contextual factors shape parental behavior toward immunization. Yet, existing research is fragmented across countries and regions, lacking a cohesive synthesis of the key determinants that influence adherence among these groups. This narrative review aimed to consolidate and critically analyze the literature on determinants of parental adherence to childhood immunization among children under five in the marginalized population in Asia. By identifying common barriers and facilitators across cultural and national contexts, this review seeks to inform culturally responsive immunization strategies and policies to improve vaccine equity across the region.

## 2. Materials and Methods

### 2.1. Study Selection

Although systematic search methods and PRISMA guidelines were used to enhance transparency and reproducibility, this review adopts a narrative design and thematic synthesis approach. We conducted a narrative literature review of studies published between 2015 and 2025 (with earlier landmark studies included if relevant), publications in English, and studies conducted in Asia, with a country-specific focus. Searches were performed in PubMed, Scopus, and Google Scholar. Search strategies were adapted to the syntax of each database, and full Boolean strings are provided in Table 1. A small number of systematic reviews were retained for contextual insights. Their findings were not coded, counted, or included in the synthesis themes. This review did not adopt an umbrella design or aggregate findings from secondary reviews.

Geographic scope of selected studies (Figure A2/Appendix B): this review focuses on marginalized populations within South and Southeast Asia, where disparities in childhood immunization coverage are most pronounced due to intersecting social, economic, and health system barriers. Countries represented in the included studies include India, Bangladesh, Nepal, Pakistan, Myanmar, Indonesia, Vietnam, Cambodia, and the Philippines.

Refugee and displaced populations such as the Rohingya (from Myanmar, residing in Bangladesh), urban slum dwellers, and left-behind children of migrant workers are considered within the national and subnational contexts of these countries.

Western Asia was excluded to maintain regional coherence, as the sociopolitical drivers of marginalization and immunization system structures differ substantially. Inclusion would have introduced heterogeneity that limits comparative synthesis across themes.

### 2.2. Eligible Criteria

The inclusion and exclusion criteria focused on determinants of parental and caregiver adherence to childhood immunization in children under five in marginalized populations across Asia. The 2015–2025 time frame was chosen to capture evidence relevant to recent immunization policies and health system changes. Only English-language studies were considered due to feasibility. Restricting to marginalized populations helped highlight barriers and facilitators among groups at greatest risk of incomplete immunization. Besides original articles, quantitative studies were included. Qualitative studies were also incorporated to provide contextual insights into barriers and facilitators. In addition to primary research articles, relevant systematic reviews were included when they were used to contextualize findings and highlight broader patterns, while individual studies provided detailed evidence from marginalized populations in Asia.

Geography: This review focused on “Asia” including South, Southeast, and East Asia, following the United Nations’ geoscheme classification.

Populations: parents, mothers, fathers, or primary caregivers responsible for vaccination decisions for children under five years of age.

Context: marginalized populations defined as groups facing barriers to healthcare access due to social, economic, cultural, or geographic disadvantages (e.g., slum dwellers, rural poor, urban poor, indigenous peoples, migrants, refugees, minorities, hard-to-reach populations, underserved or disadvantaged groups).

We excluded studies on adult immunization, clinical vaccine efficacy trials, and studies focusing exclusively on a single vaccine (e.g., measles-only, polio-only). The aim of this review was to identify determinants of parental adherence to the overall childhood immunization schedule under the Expanded Program on Immunization (EPI), rather than determinants specific to one antigen. An exception was made when such studies provided insights into broader determinants of parental adherence (e.g., socioeconomic status, cultural beliefs, access barriers) that influence the entire childhood immunization schedule. In these cases, determinants were extracted and synthesized at the program level rather than vaccine-specific. Countries of Western Asia, including those in the Arabian Peninsula and the broader Middle East and North Africa (MENA) region, were excluded.

### 2.3. Search Strategy

This narrative review was performed according to the guidelines of Preferred Re-porting Items for Systematic Reviews and Meta-Analyses (PRISMA).The search was conducted from 3 January 2025 to 15 May 2025.Relevant studies were identified from the major bibliographic databases (PubMed, Scopus, Google Scholar) that are presented in Table A1/Appendix A, focusing on determinants of parental adherence to childhood immunization in children under five in marginalized populations in Asia. The comprehensive search string of published literature applied the combinations of key terms and Boolean operators (see Table 1).

**Figure 1:**
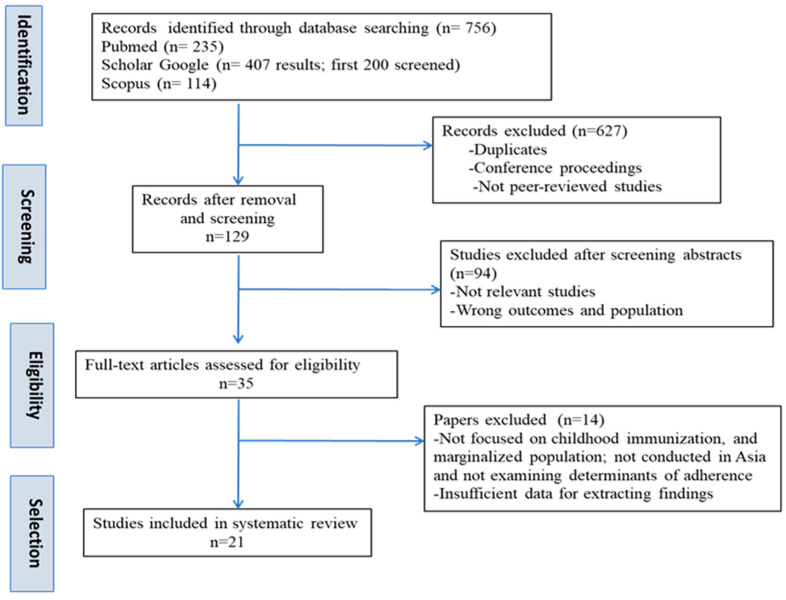
The PRISMA flow diagram describing the study selection process. Note: This flow diagram follows PRISMA-style formatting for transparency but reflects procedures adapted to a narrative literature review.

The PRISMA diagram (Figure 1) reveals the selection process and shows the reasons for exclusion. The initial search across PubMed, Scopus, and Google Scholar yielded 756 records. After removing duplicates, conference proceedings, and non-peer-reviewed sources (*n*) = 627), 129 records remained for title and abstract screening. Of these, 94 records were excluded for reasons including the following: wrong population (not parents/caregivers of under-five children, or not marginalized groups), wrong setting (studies conducted outside Asia or in high-income countries), wrong outcome/focus (articles on vaccine efficacy, safety, or clinical outcomes rather than determinants of parental adherence), and publication type/insufficient data (commentaries, editorials, protocols, or abstracts lacking relevant information).Thirty-five full-text articles were assessed for eligibility. Fourteen were excluded for the same reasons—wrong population *(n* = 5), wrong outcome *(n* = 4), wrong setting *(n* = 2), adult immunization focus *(n* = 2), and insufficient data *(n* = 1). Finally, 21 studies met all inclusion criteria and were included in the narrative synthesis. Two included studies were themselves systematic reviews.These were not integrated into the thematic synthesis alongside primary studies.Instead, they were retained for context and triangulation.Findings from these reviews were cited descriptively where relevant but were not used to identify or code determinants.No data were duplicated from primary studies already included.

### 2.4. Data Extraction and Analsis

Findings from quantitative studies were summarized to highlight measurable determinants such as socioeconomic, demographic, and healthcare access factors, while qualitative studies were narratively integrated to capture contextual influences.Four authors independently extracted data using a standardized template in Microsoft Excel, and qualitative findings were organized and coded using NVivo 12 to ensure consistency and reduce bias.The extracted information includedAuthor, Year; Country/Region; Population (parents/caregivers/children); Study Design; Sample size/Data source; Determinants examined; Key findings (direction/association); Qualitative themes/quotes; Contextual insights (policy, system, cultural); and Strengths/Limitations (see Table 2). The primary outcome variables were the determinants of parental adherence to childhood immunization.Discrepancies between extractors were resolved through discussion and consensus, with adjudication by a senior author when necessary.For reference management, EndNote X9 was used to organize citations and remove duplicates.The evidence was then synthesized thematically to identify converging and complementary influences across study types.

### 2.5. Risk of Bias and Quality Assessment

Because this review included both quantitative and qualitative studies, formal meta-analysis was not feasible. Instead, study quality was appraised descriptively. For quantitative studies, considerations included clarity of population definition, representativeness of sampling, validity of immunization outcome measures, and adjustment for confounding factors (adapted from the Newcastle–Ottawa Scale) [24]. For qualitative studies, credibility and transferability of findings were assessed based on methodological clarity and depth of contextual insights (adapted from the CASP Qualitative Checklist) [25]. For review-level evidence, transparency of search strategies, inclusion criteria, and synthesis methods were considered. In Table A2/Appendix A, of the twenty-one included studies, nine were judged to be at low risk of bias, primarily large-scale quantitative analyses based on nationally representative datasets such as DHS, MICS, and IFLS, as well as two systematic reviews. Only primary studies included in thematic synthesis were appraised for risk of bias.

The remaining twelve studies were assessed as moderate risk, consisting mainly of smaller cross-sectional surveys, qualitative inquiries, and mixed-methods designs that, while valuable for contextual insights, were limited by sample size, non-probability sampling, or restricted generalizability. No study was classified as high risk, and all provided sufficient methodological transparency to contribute meaningfully to the synthesis. Given the narrative design of this review, no study was excluded on the basis of quality alone, but risk-of-bias assessments were used to guide interpretation of findings.

## 3. Results

### 3.1. Overview of Included Studies

This review included 21 studies published between 2016 and 2025, conducted in India, Bangladesh, Pakistan, Nepal, Vietnam, China, Malaysia, Lao PDR, and the Philip-pines, along with two regional systematic reviews from Southeast Asia. Designs included quantitative cross-sectional surveys [4,5,6,17,19,20,21], large national analyses [7,8,15,16], qualitative studies [3,9,18,22,23,24], mixed-methods approaches [10,11], and systematic reviews [12,13,14]. Populations studied were slum dwellers, refugees, migrants, left-behind children, ethnic minorities, and rural/remote households. Data extractions from included studies are described in Table 2.

### 3.2. Thematic Synthesis of Determinants of Parental Adherence

A narrative thematic synthesis was conducted to identify recurring determinants of parental adherence across included studies. Themes were developed through an inductive–deductive approach, combining a priori coding (based on the research objectives and literature) with emergent codes identified during full-text review). Coded segments were clustered into five overarching thematic domains:

(1) Socioeconomic and access inequities;
(2) Trust, cultural beliefs and social norms;
(3) Migration, refugee, and mobility-related vulnerabilities;
(4) Household and caregiver dynamics;
(5) Health system and program.

Representative codes and illustrative quotes from qualitative studies are presented in Table 3 to support transparency and contextual insight.

**Figure A1:**
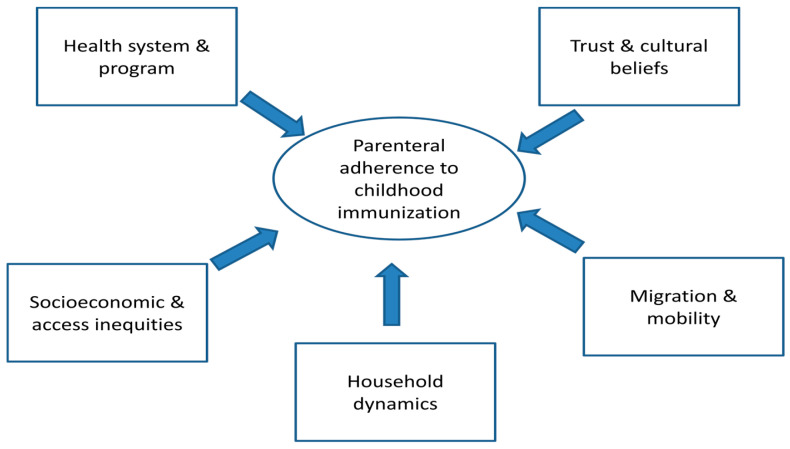
Conceptual Framework for determinants of parental adherence to childhood immunization in marginalized populations (Asia).

**Figure A2:**
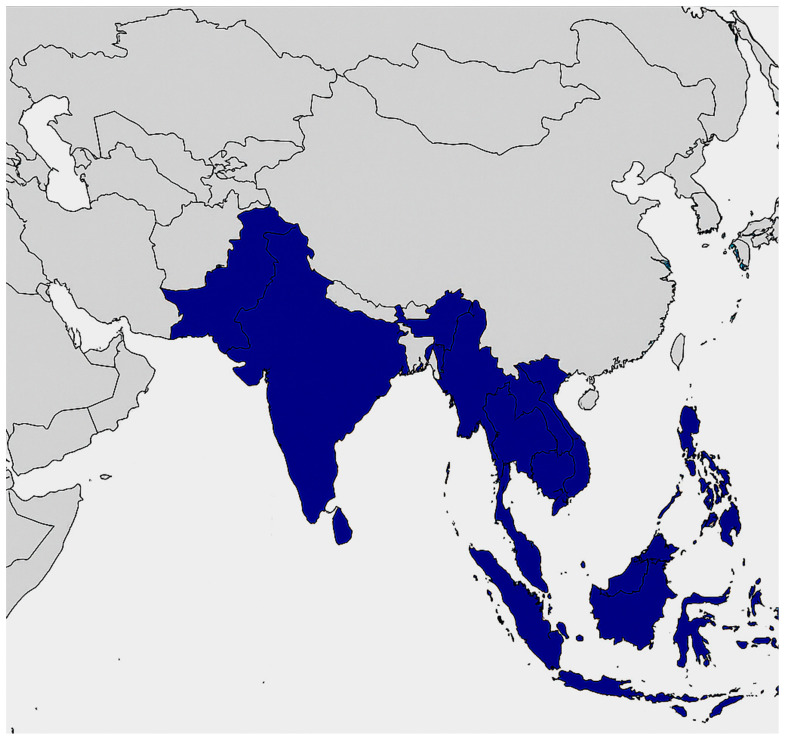
Countries included in the review (South and Southeast Asia). Note: Countries highlighted in blue represent those within South and Southeast Asia that were included in this review. Countries shown in gray (e.g., in Western Asia and the Middle East) were excluded from the review due to regional, political, and health system differences that limit thematic comparability.

Mapping of determinants to thematic domains is provided in Table A3/Appendix A. The interactions between five domains are visualized in Figure A1/Appendix B.

#### 3.2.1. Socioeconomic and Access Inequities

Socioeconomic position consistently shaped parental adherence. Maternal education and household wealth were repeatedly shown to increase the likelihood of complete immunization [5,8,12]. For instance, Bangladesh surveys revealed that possession of an EPI card and higher maternal education reduced zero-dose status in slums and haor communities [5]. In India, national analyses confirmed that facility quality interacts with household wealth, exacerbating rich–poor disparities in rural areas [7]. In Indonesia, inequalities by maternal education and socioeconomic status persisted across provinces [15]. Geographic access compounded socioeconomic barriers. Remote and ethnic minority populations in the Lao PDR and Vietnam border and highland regions experienced lower adherence due to distance, transport costs, and language barriers [3,4,16].

#### 3.2.2. Trust, Cultural Beliefs, and Social Norms

Trust in healthcare providers and cultural beliefs were major determinants of parental decisions. In Pakistan’s slums, adherence was hindered by misconceptions about side effects and religious opposition [11]. In Bangladesh’s Rohingya settlements, parents in registered camps demonstrated higher adherence than those in makeshift camps, reflecting stronger trust in formal systems [17].

Social norms and interpersonal communication strongly predicted vaccination intentions in India’s urban slums, explaining nearly half of the variance in behavioral intention models [6]. In the Philippines, the Dengvaxia^®^ vaccine controversy eroded trust, reducing measles vaccine uptake [18]. A systematic review also highlighted how misinformation, online narratives, and fear of autism reinforced distrust, particularly among higher-SES and more educated parents [14].

In Nepal, caregivers described providers as unresponsive, while health workers described inadequate community cooperation, underscoring the cycle of mutual distrust [9].

#### 3.2.3. Migration, Refugee, and Mobility-Related Vulnerabilities

Refugee and migrant status was consistently linked with incomplete immunization. Among Rohingya children in Malaysia, only 2.5% were fully immunized, with birthplace and access to health services as major predictors [19]. Similarly, stateless children of North Korean refugees in China had coverage ranging only from 12% to 98%, compared with nearly universal coverage among local children [20].

Left-behind children in China had the lowest non-national immunization program (NIP) vaccine coverage, reflecting disrupted care arrangements [21]. A systematic review confirmed that left-behind children are also at greater risk of incomplete immunization and other adverse health outcomes [13]. In Vietnam’s border regions, minority children had extremely low adherence (18.9%), which was associated with structural barriers and low parental self-efficacy [4].

#### 3.2.4. Household and Caregiver Dynamics

Caregiver characteristics and family structures significantly were associated with adherence. Maternal occupation and mobility were associated with incomplete immunization in India, where working mothers were less able to attend vaccination sessions [10]. In Bangladesh’s refugee camps, father’s education and employment predicted adherence, particularly in makeshift settlements [17]. In Vietnam’s border areas, firstborn and younger children were more likely to be vaccinated [4]. Extended family members also played a role. Grandmothers and fathers were actively engaged in decision-making in Nepal [9], while tribal chiefs were gatekeepers in rural Pakistan, shaping community-level acceptance [22].

#### 3.2.5. Health System and Programmatic Factors

Supply-side determinants emerged as equally important. In India, higher-quality facilities improved both the completeness and timeliness of vaccination [7]. In Pakistan’s underserved districts, shortages of female vaccinators, poor staff capacity, and weak community engagement contributed to dropout and refusals [22]. Provider perspectives from China emphasized systemic barriers, including staff shortages, vaccine hesitancy, and fragmented information systems [23].

Programmatic tools also mattered. EPI card possession was one of the strongest predictors of adherence in Bangladesh [5]. In China, providers recommended improved follow-up mechanisms such as maternal–child health visits and school verification to strengthen continuity [23]. Reviews across Southeast Asia confirmed the positive impact of antenatal care visits, institutional delivery, and maternal–child health integration on immunization compliance [12].

#### 3.2.6. Summary

Across the 21 studies, adherence to childhood immunization among marginalized populations in Asia was consistently shaped by structural inequities (education, wealth, geography), trust and social perceptions, migration-related vulnerabilities, household decision-making dynamics, and health system/programmatic quality. These determinants interact and reinforce one another, highlighting the need for equity-oriented, context-sensitive strategies to strengthen EPI coverage in marginalized populations.

## 4. Discussion

### 4.1. Principal Findings

This review synthesized 21 studies on determinants of parental adherence to childhood immunization among children under five in marginalized populations in South, Southeast, and East Asia. Across settings, adherence was shaped by five interrelated themes.

Socioeconomic and access inequities were consistent determinants. Maternal education, household wealth, and possession of vaccination documentation (e.g., EPI card) strongly predicted adherence. Remoteness and transport costs compounded inequities, particularly for highland, border, and minority communities.

Parental decisions regarding childhood immunization were shaped by trust, cultural beliefs, and prevailing social norms. Misconceptions about vaccine safety, religious objections, and misinformation reduced uptake, whereas trust in providers and supportive social norms enhanced it. The Dengvaxia^®^ controversy in the Philippines demonstrated how one event can erode trust across the broader immunization program.

Migration, refugee, and mobility-related vulnerabilities were striking. Refugee and stateless children in Malaysia and China, and left-behind children of migrants, had markedly lower coverage compared to host populations. Mobility and weak registration systems undermined continuity of care.

Household and caregiver dynamics were important contextual factors. Fathers, grandmothers, and community leaders were often reported to play key roles in parental decision-making, underscoring that vaccination is not solely a maternal responsibility. Larger household size, birth order, and maternal employment status were also associated with immunization uptake.

Finally, health system and programmatic factors strongly shaped adherence. Facility readiness, staffing, provider communication, and follow-up systems were frequently associated with immunization inequities.

Tools such as the EPI card in Bangladesh emerged as powerful equity instruments, while lack of outreach, female vaccinators, and community mobilization undermined program success.

### 4.2. Comparison with Prior Reviews

The findings of this review are consistent with earlier global analyses that identified maternal education, socioeconomic status, and health service access as core determinants of immunization coverage [26,27,28]. In this review, “coverage” refers to the proportion of children who received vaccinations, commonly used as a health system performance indicator. “Adherence”, on the other hand, refers to caregivers’ timely compliance with the recommended vaccination schedule—capturing not just whether vaccines were received but also whether they were administered at the appropriate ages and intervals.

Like prior reviews, this synthesis confirms that education and wealth gradients are strongly associated with child vaccination status across diverse contexts.

However, the present review adds four contributions. First, it emphasizes context-specific vulnerabilities unique to Asia, such as left-behind children of migrants, ethnic minorities in highlands and border areas, and the lasting effects of vaccine controversies. Second, it demonstrates the interdependence of demand- and supply-side determinants. For instance, demand may be high but undermined by provider distrust, weak infrastructure, or lack of follow-up. Similarly, possession of an EPI card was consistently associated with improved adherence, highlighting the role of programmatic design. Trust, provider relationships, and communication are equally critical. For example, even when awareness was high, distrust of providers or poor facility quality limited adherence—a pattern not fully captured in quantitative-only global reviews. Third, this review underscores how vaccine controversies and misinformation uniquely shape adherence in Asia. The Dengvaxia^®^ case in the Philippines illustrates how negative experiences with one vaccine can spill over to others, echoing findings from global research on how rumors and misinformation erode vaccine confidence [18,29,30].

Finally, unlike broader global reviews that often emphasize national averages, this synthesis focuses explicitly on marginalized populations. In doing so, it highlights how structural exclusion—systemic barriers embedded in health, social, and legal systems—amplifies inequities in immunization. Such exclusion arises when refugee and stateless children are denied access due to legal restrictions, when migrants and left-behind children are missed because of weak registration and follow-up systems, or when ethnic minorities in remote areas face geographic and language barriers not addressed by mainstream programs. These dynamics show that under-immunization is not merely the result of parental choice but often reflects institutional and societal structures that fail to equitably serve all groups [31].

### 4.3. Implications for Policy and Practice

Improving immunization adherence in marginalized populations requires integrated strategies. Equity-focused approaches should prioritize underserved groups by expanding outreach, reducing indirect costs, and strengthening registration systems, consistent with global recommendations to target zero-dose children [32].

Community engagement must rebuild trust, counter misinformation, and include fathers, grandmothers, and community and religious leaders—an approach supported by other reviews showing that community ownership improves vaccine uptake [33].

The integration of maternal and child health (MCH)services with routine immunization is recognized as an important global health strategy, particularly in low- and middle-income countries. Linking immunization with services such as antenatal care, institutional delivery, and school-entry checks can reduce missed opportunities and improve continuity of care. This review reinforces prior evidence showing that integrated service delivery not only strengthens immunization uptake but also enhances equity by reaching mothers and children who might otherwise remain outside the health system [34].

Migrant and refugee inclusion is essential, echoing calls from global health policy frameworks that immunization services should transcend legal or administrative barriers [35].

### 4.4. Evidence Gaps and Future Research Directions

Despite the breadth of evidence, several gaps remain. Populations such as indigenous communities beyond Vietnam, seasonal migrants, fathers as primary decision-makers, and caregivers of children with disabilities were underrepresented. Determinants related to digital registration/ID systems, disinformation dynamics in Asian contexts, interpersonal provider–caregiver quality, and indirect costs remain poorly studied.

Furthermore, programmatic evidence is particularly limited. Few studies rigorously evaluated outreach/mobile services, incentive schemes, defaulter tracing, or multilingual counseling, despite their relevance to marginalized groups. Future research must move beyond descriptive studies to rigorous intervention evaluations, as emphasized in recent global reviews [36].

### 4.5. Strengths and Limitations

This review contributes a comprehensive synthesis across multiple marginalized populations in Asia, integrating evidence from surveys, and qualitative inquiries. Its focus on determinants rather than vaccine efficacy highlights the social and structural barriers to immunization adherence. Study quality was considered in interpreting the results.Findings from studies rated as lower quality (e.g., lacking clarity in methods or sampling) were interpreted with caution and weighed less heavily in the synthesis of themes.Two retained systematic reviews served a supplementary role.Their findings were referenced contextually but did not inform the thematic structure or determinant mapping to avoid duplication bias.

However, limitations include restriction to English-language studies between 2015 and 2025, which may exclude local-language research. The narrative approach precludes meta-analysis and introduces potential selection bias. Study heterogeneity further limited comparability across countries.

## 5. Conclusions

Parental adherence to childhood immunization in marginalized Asian populations is shaped by (1) socioeconomic inequities, (2) trust and cultural beliefs, (3) migration vulnerabilities, (4) household dynamics, and (5) health system quality. These determinants interact, reinforcing disadvantages. Addressing them will require equity-oriented, context-specific approaches that integrate service delivery, strengthen trust, and adapt to the realities of marginalized groups. Filling evidence gaps with targeted research and program evaluations is essential to achieve universal immunization coverage and ensure no child is left behind.

## Figures and Tables

**Table 1:** Key terms or Boolean operators used for searching in each database.

Search	Search Terms (Boolean Operators)
Pubmed	((“child” [Title/Abstract] OR “children” [Title/Abstract] OR “under-five” [Title/Abstract] OR “infant *” [Title/Abstract]) AND (“immunization” [Title/Abstract] OR “immunisation” [Title/Abstract] OR “vaccination” [Title/Abstract] OR “vaccine coverage” [Title/Abstract] OR “vaccine adherence” [Title/Abstract] OR “vaccination compliance” [Title/Abstract]) AND (“determinant *” [Title/Abstract] OR “factor *” [Title/Abstract] OR “predictor *” [Title/Abstract] OR “barrier *” [Title/Abstract] OR “facilitator *” [Title/Abstract] OR “enabler *” [Title/Abstract]) AND (“marginalized” [Title/Abstract] OR “minority” [Title/Abstract] OR “migrant” [Title/Abstract] OR “refugee” [Title/Abstract] OR “indigenous” [Title/Abstract] OR “slum” [Title/Abstract] OR “underserved” [Title/Abstract] OR “low-income” [Title/Abstract] OR “disadvantaged” [Title/Abstract]) AND (“Asia” [MeSH Terms] OR “India” [MeSH Terms] OR “Bangladesh” [MeSH Terms] OR “Nepal” [MeSH Terms] OR “Pakistan” [MeSH Terms] OR “Myanmar” [MeSH Terms] OR “Vietnam” [MeSH Terms] OR “Thailand” [MeSH Terms] OR “Indonesia” [MeSH Terms] OR “Philippines” [MeSH Terms] OR “China” [MeSH Terms]))
Scopus	TITLE-ABS-KEY ((child * OR “under-five” OR infant *) AND (immunization OR immunisation OR vaccination OR “vaccine coverage” OR “vaccine adherence” OR compliance) AND (determinant * OR factor * OR predictor * OR barrier * OR facilitator * OR enabler *) AND (marginalized OR minority OR migrant OR refugee OR indigenous OR slum OR underserved OR disadvantaged) AND (Asia OR India OR Bangladesh OR Nepal OR Pakistan OR Myanmar OR Vietnam OR Thailand OR Indonesia OR Philippines OR China)
Google Scholar	(“child” OR “children” OR “under five” OR “infant” OR “toddler”) AND (“immunization” OR “immunisation” OR “vaccination” OR “vaccine coverage” OR “vaccine adherence” OR “vaccination compliance”) AND (“determinant” OR “factor” OR “predictor” OR “barrier” OR “facilitator” OR “enabler” OR “vaccine confidence” OR “social norms” OR “trust in health system”) AND (“marginalized” OR “minority” OR “migrant” OR “refugee” OR “indigenous” OR “slum” OR “urban poor” OR “rural poor” OR “left-behind” OR “underserved” OR “disadvantaged” OR “hard-to-reach”) AND (“Asia” OR “India” OR “Bangladesh” OR “Pakistan” OR “Nepal” OR “Vietnam” OR “Myanmar” OR “Thailand” OR “Indonesia” OR “Philippines” OR “China” OR “Malaysia”)

Note: The asterisk (*) is a truncation symbol used in database searches to include variations of a word (e.g., infant, infants, infantile).

**Table 2:** Data extraction from 21 studies included in the systematic review.

Author, Year	Country/Region	Population (Parents/Caregivers/ Children)	Study Design	Sample Size/Data Source	Determinants Examined	Key Findings (Direction/ Association)	Qualitative Themes/Quotes	Contextual Insights (Policy/System/ Cultural)	Strengths/Limitations
Nguyen et al., 2025 [3]	Vietnam (Daklak highlands)	Ethnic minority mothers and HCWs	Qualitative (IDIs, FGDs, observation)	25 communes, 9 districts	Socioeconomic vulnerability, social networks, trust in HCWs, structural marginalization	Acceptance shaped by poverty and marginalization. Trust in HCWs fostered acceptance; distrust linked to isolation.	Isolation → distrust; social network support → adherence.	Structural vulnerability explains outbreaks despite reported coverage.	Strong ethnographic design; limited sample generalizability.
Tran et al., 2025 [4]	Vietnam–Cambodia border [Dong Thap]	Minority parents of children <5 years	Quantitative (Survey+ regression)	449 parents	Child age, birth order, perceived barriers, self-efficacy	Adherence 18.9%. Younger and first born ↑ adherence; high barriers ↓ adherence; high self-efficacy ↑ adherence.	N/A	HBM framework highlights self-efficacy and barriers; strong disparities at borders.	Robust sample; cross-sectional design limits causality.
Das et al., 2024 [5]	Bangladesh (slums and haors)	Caregivers of 4.5–23 m children	Quantitative (LQAS survey)	504 households, 18 clusters	Zero-dose, EPI card, maternal age/education, household earning	32%ZD/UI overall; 59%in slums, 32%in haors. EPI card strongest predictor; maternal age/education mattered.	N/A	EPI card availability critical equity tool.	Good LQAS design; localized findings.
Rimal et al., 2024 [6]	India (Varanasi slums)	Caregivers of <2 years	Quantitative (survey)	2058 interviews	Social norms, vaccine confidence, interpersonal communication	All predictors associated with intention; interaction effects significant; explained 46% variance.	N/A	Social norms and communication. amplify/attenuate vaccine confidence.	Large survey; intention not behavior.
Summan et al., 2022 [7]	India (rural, multi-state)	Children <2 years, households across 24 states	Quantitative (national survey analysis, decomposition)	44,571 households, 1346 facilities	Facility quality, infrastructure, service delivery, socioeconomic status	Higher facility quality linked with full immunization and timeliness; gaps larger among poor households.	N/A	Strengthening rural facility quality can close rich-poor gaps.	Robust national survey and facility data; limited causal inference.
Siramaneerat & Agushybana, 2021 [8]	Indonesia	Mothers and their children aged 12–24 months	Multilevel analysis of 2017 Indonesia Demographic and Health Survey (IDHS)	4753 children	Child-level: sex, age, birth order. Parent-level: maternal age at delivery, maternal and paternal education, father’s occupation, maternal occupation, antenatal care (ANC). Community-level: urban/rural, region, proportion of public health centers (PHCs).	58.2%fully immunized. Children of higher birth order, older mothers, and educated parents more likely to be fully vaccinated. ANC positively associated. Wealthier households and urban areas had higher coverage. Communities with more PHCs had better coverage.	N/A	Large disparities across regions; remote and rural children face greater barriers. Strengthening PHCs and midwifery services is essential.	Strengths: nationally representative data, multilevel analysis. Limitations: recall bias possible, timing of vaccination not analyzed, regional heterogeneity limits direct policy transfer.
Paul et al., 2022 [9]	Nepal (Makwanpur District)	Mothers, fathers, grandmothers, FCHVs, HCWs, govt reps	Qualitative (IDIs, KIIs, FGDs)	76 participants (54 mothers, 5 fathers, 5 grandmothers, 12 HCWs/govt reps)	Knowledge, social norms, trust, provider support, infrastructure	High awareness and demand, but lack of mutual trust between caregivers and providers a key barrier. Supply issues: poor infrastructure, low support for HCWs.	Distrust between providers and caregivers; HCWs cite inadequate support.	Trust critical; interventions should address both social and structural barriers.	Rich qualitative depth; district-level scope only.
Francis et al., 2021 [10]	India (Vellore, Tamil Nadu)	Children 12–23 months in disadvantaged communities	Mixed-methods (survey + FGDs)	100 households + 43 parents in FGDs	Maternal occupation, mobility, awareness, misinformation	65–77%fully vaccinated. Working mothers less likely to vaccinate; mobility and misinformation barriers.	FGDs: mobility issues, myths hinder uptake.	Targeted outreach needed for tribal groups; Mission Indradhanush partly effective.	Small survey sample; rich FGDs.
Muhammad et al., 2023 [11]	Pakistan (Karachi slums)	Parents/caregivers of children 12–23 months	Mixed-methods (survey + IDIs)	840 households, 412 children	Fear of side effects, social/religious influences, lack of awareness, misconceptions	Only 49% fully vaccinated. Misconceptions and religious barriers common. Mapping showed fragmented interventions.	Fear of side effects; religious misconceptions reported in IDIs	Need for coordinated demand-generation strategies across stakeholders.	Large slum survey, but cross-sectional and self-report limitations.
Kalaij et al., 2021 [12]	Southeast Asia (multi-country)	Children and caregivers	Systematic review	16 observational studies, 41,956 subjects	Maternal age, education, occupation, SES, antenatal care, family size	Compliance ↑ with older maternal age, higher SES, antenatal care, parents in health/govt jobs. Noncompliance ↑ with younger age, large families, poverty, low education.	N/A	Confirms classic determinants across SEA; evidence base observational, gaps in marginalized groups.	SR strength; heterogeneity and limited focus on marginalized populations.
Racaite et al., 2021 [13]	Multi-country (review, China focus)	Left-behind children	Systematic review	34 studies	Parental migration, caregiver absence	Left-behind children had more incomplete immunization, stunting, risky behaviors.	Caregiver absence noted in multiple studies.	Migration creates risk profiles beyond immunization.	Evidence concentrated in China; few other Asian countries.
Novilla et al., 2023 [14]	Multi-countries (MMR focus, lessons applicable)	Parents/caregivers	Systematic review	115 articles (2000–2022)	Education, income, race, religiosity, political affiliation, social networks, trust, misinformation	Fear of autism most cited reason for hesitancy. Hesitancy clustered in higher-SES, educated parents preferring online/social media narratives.	N/A	Hesitancy socially patterned; interventions must target misinformation and rebuild trust.	Comprehensive SR; most evidence, indirect transferability.
Sinuraya et al., 2024 [15]	Indonesia	Children aged 1–14 years (IFLS dataset, analyzed at household level with parent/caregiver characteristics)	Quantitative (secondary data analysis of IFLS-5, logistic regression)	*n*= 16,236 children across 13 provinces	Maternal education, maternal antenatal/postnatal care, maternal tetanus immunization, father’s age, parental health insurance, household size, wealth index, religion, urban/rural residence, region, parental media access, smoking status, place of delivery	- Higher maternal education, maternal tetanus immunization, urban residence, health insurance, and wealth associated with complete immunization. - Father’s age 41–50 years linked with higher adherence. - Larger families associated with better immunization (contrary to some literature).	Not applicable (quantitative only)	Strong national survey; evidence of socio-economic, regional, and parental health behavior influences. Highlights Indonesia’s persistent gaps despite NIP coverage.	Strengths: large nationally representative dataset; advanced statistical modeling with multiple imputation. Limitations: secondary data, recall bias from parental reporting, missing data (imputation used).
Ichimura et al., 2022 [16]	Lao People’s Democratic Republic	Caregivers of children aged 12–35 months	Nationwide cross-sectional study (multistage cluster sampling)	256 child–caregiver pairs (out of 416 targeted); national seroepidemiological survey 2019	Place of birth, place of vaccination, residence type (fixed/mobile), family size, maternal/paternal education, occupation, ethnicity, access to health facility, outreach vaccination	67.6% fully immunized. Hospital/health facility birth strongly associated with full immunization (AOR 9.75). Outreach vaccination in village negatively associated.Fixed residence associated with better coverage. Missing doses mainly Hep B at birth, PCV, and measles.	N/A	Ethnic minorities and remote areas remain underserved. Outreach/mobile services may help, but quality and trust issues persist.	Documented immunization records, national sampling. Limitations: exclusion of children without records, potential under-representation of ethnic minorities, cross-sectional design
Ahmed et al., 2023 [17]	Bangladesh (Cox’s Bazar)	Rohingya refugee parents	Quantitative (cross-sectional survey)	224 parents	Knowledge, camp type, parental education/employment	63% completed immunization. Knowledge and registered camp residence increased adherence; father’s education/employment mattered.	N/A	Disparities between registered vs. makeshift camps; highlights importance of health education.	Good sample in difficult-to-access refugee camps; limited generalizability beyond Cox’s Bazar.
Miras, Regencia & Baja, 2023 [18]	Philippines (Pasay City, Metro Manila)	Parents/caregivers and healthcare workers; children under 5 indirectly affected through caregivers	Qualitative study	*n*= 41 participants (26 parents, 9 health staff, 6 mothers in FGD)	Trust, misinformation, media influence, healthcare worker communication, political context, social norms	- Dengvaxia^®^ controversy caused mistrust and hesitancy toward measles vaccination. - Media sensationalism, - HCWs had limited information and struggled to reassure parents	Quotes illustrate fear, anger, and mistrust	Demonstrates how a vaccine crisis (Dengvaxia^®^) undermined confidence in broader immunization. Importance of transparent communication and trust-building.	Strengths: rich qualitative insights; theory-driven analysis Limitations: single-site, urban setting; limited generalizability; purposive sample.
Al-Haroni et al., 2023 [19]	Malaysia (Ampang district, Kuala Lumpur)	Rohingya refugee children (school-age, 3–14 yrs)	Quantitative (cross- sectional)	243 children (guardians surveyed)	Child’s age, place of birth, access to healthcare	2.5%fully immunized; significant determinants = birth country, age, access to healthcare.	N/A	Refugees face systemic barriers; NGO/UNHCR programs insufficient.	Important refugee sample; limited to one district.
Chung et al., 2016 [20]	China (Yanbian)	Children born to North Korean refugee mothers	Quantitative (survey + vaccination cards)	Refugee vs. Chinese/migrant children	Father’s age, sibling presence, legal status	Coverage 12–98% vs. ~99%Chinese children. Father’s age and siblings ↓ vaccination.	N/A	Stateless children excluded from health system; legal invisibility critical barrier.	Comparative survey; limited to one prefecture.
Zhou et al., 2023 [21]	China (Zhejiang & Henan)	Migrant and left-behind families	Quantitative (cross-sectional survey)	1648 caregivers	Coverage, knowledge, satisfaction, migration status	Non-NIP coverage highest in locals, then migrants, lowest in left-behind. Satisfaction key determinant.	N/A	Non-NIP disparities show hidden inequities beyond EPI.	Large survey; self-report bias possible.
Qayyum et al., 2021 [22]	Pakistan (Rajanpur district)	Rural/tribal caregivers and providers	Qualitative (IDIs, FGDs)	24 IDIs, 7 FGDs	Health system barriers, female staff, engagement, social mobilization	Acceptability varied: refusal, drop-out. Lack of female vaccinators, poor engagement, reliance on tribal chiefs.	Poor mobilization; reliance on tribal chiefs; demand fluctuated.	Weak community mobilization and system support undermine coverage.	Qualitative depth; not generalizable quantitatively.
Lin et al., 2022 [23]	China (Sichuan, Guangdong, Henan)	Providers (vaccinators, HCWs)	Qualitative (26 interviews)	26 providers	Provider perceptions: child factors, caregiver SES/education, institutional issues	Coverage lower among migrants/left-behind. Barriers: staffing, info systems, hesitancy.	Providers cite staff shortages, poor follow-up systems.	Recommendations: integrate MCH visits, family follow-up, school verification.	Good provider perspective; small sample.

Note: ↑ indicates an increase; ↓ indicates a decrease.

**Table 3:** Representative codes and illustrative quotes and insights supporting thematic domains.

Themes	Codes	Illustrative Quotes/Insights
Trust, cultural beliefs and social norms	Mistrust in government	“Parents were afraid the vaccines were unsafe after what happened with Dengvaxia.” (Philippine) [18]
	Traditional beliefs	“Some parents believe herbal treatments are safer than injections.”(Vietnam) [3]
	Religious norms	“Imams in our village told us vaccines are not halal.” (Pakistan) [11]
Househod and caregiver dynamics	Patriarchal decision-making	“My husband decides whether our child gets vaccinated” (Vietnam) [3]
	Maternal employment barriers	“I had no time to take him for the shot because I work all day” (Pakistan) [22]
	Grandparent caregiving	“My mother-in-law thought the child had enough vaccine already.” (Philippine) [18]
Migration, refugee, and mobility-related vulnerabilities	Statelessness	* Lack of legal documentation (such as ID cards) was cited as a key barrier to accessing immunization services. [19]
	Caregiver discontinuity	“My child lives with my sister, and she forgot the schedule.” (Pakistan) [22]
	Mobile population	“We move often for work, so it is hard to track appointments.” (India) [10]
Health system and program.	Provider-caregiver trust gap	“There’s still a lot of distrust between caregivers and providers. People hesitate when there’s no familiar face.” (Nepal) [9]
	Health worker support	“Our health workers don’t get the support they need to reach all households.” (Nepal) [9].
	Gender barrier in staff	“Some mothers refused because there was no female vaccinator available.” (Pakistan) [22]
	Weak community mobilization	“If the tribal chief does not support it, people don’t come.” (Pakistan) [22].
Socioeconomic and access inequities	Poverty and rural isolation	* Caregivers in remote areas often face long travel distances, making vaccination logistically difficult. (Lao PDR) [16] “Demand fluctuated. Some areas never received mobilization support.” (Pakistan) [22]
	Education and awareness	“Coverage was much lower in communities where caregivers had limited schooling or health knowledge.” (China) [23]
	Provider perceptions of caregiver socioeconomic status	“Staff say caregivers who are poor or left-behind tend to miss doses more often.” (China) [23].

Note: Quotes marked with an asterisk (*) represent paraphrased insights derived from quantitative or mixed-methods studies. These are not direct participant quotations but are included to illustrate key themes where qualitative evidence was limited.

## Data Availability

Data generated in this study is available by contacting the corresponding author, Cua Ngoc Le, if requested reasonably.

## Appendix A

**Table A1:** Database and source used in review.

Database/Source	Coverage	Notes
PubMed/MEDLINE	Biomedical and public health literature	MeSH + free text
Scopus	Multidisciplinary, large coverage	Includes conference proceedings
WHO Global Index Medicus (WPRIM, IMSEAR, AIM)	Regional databases for Asia	Captures local research
Google Scholar	General search and gray literature	First 200 results screened (out of 407 retrieved)

**Table A2:** Risk of bias and quality appraisal of included studies.

Author, Year	Country/Region	Study Type	Appraisal Outcomes	Notes
***** Ichimura et al., 2022 [16]	Lao People’s Democratic Republic	Nationwide cross-sectional study (multistage cluster sampling)	Low risk	Nationwide sampling; vaccination records validated; minor bias from excluding children without cards.
***** Siramaneerat & Agushybana 2021 [8]	Indonesia	Quantitative DHS multilevel analysis	Low risk	Large representative DHS dataset; multilevel modeling; recall bias possible.
***** Sinuraya et al. 2024 [15]	Indonesia	Quantitative IFLS secondary analysis	Low risk	Large national dataset; advanced regression; limited by secondary data use.
***** Miras et al., 2023 [18]	Philippines	Qualitative ethnography	Moderate risk	Rich contextual insights; theory-driven; limited by single urban setting and purposive sample.
***** Das et al., 2024 [5]	Bangladesh (slums and haors)	Quantitative, cross-sectional	Moderate risk	Good description; convenience sampling; risk of reporting bias.
***** Nguyen et al., 2025 [3]	Vietnam (Daklak highlands) Ethnic minority mothers and HCWs	Qualitative (IDIs, FGDs, observation)	Moderate risk	Valuable contextual insights; small sample; limited generalizability.
***** Rimal et al., 2024 [6]	India (Varanasi slums)	Quantitative (survey)	Low risk	Large sample size. Clear determinants identified; non-probability sampling; recall bias.
***** Francis et al., 2021 [10]	India (Vellore, Tamil Nadu)	Mixed-methods (survey + FGDs)	Moderate risk.	Combines survey and FGDs; limited sample; good contextual triangulation
***** Muhammad et al., 2023 [11]	Pakistan (Karachi slums)	Mixed-methods	Moderate risk.	Sample adequate; limited confounder control; self-reported immunization status.
***** Racaite et al., 2021 [13]	Multi-country (review, China focus)	Quantitative study	Low risk	Good sampling; clear analysis; strong data on NIP vs. non-NIP vaccines.
Kalaij et al., 2021 [12]	Southeast Asia (multi-country)	Systematic review	Low risk	Transparent synthesis; PRISMA-based; complements primary studies
***** Tran et al., 2025 [4]	Vietnam–Cambodia border (Dong Thap)	Quantitative (survey + regression)	Moderate risk	Good integration of quantitative and qualitative data; limited by local scope.
***** Ahmed et al., 2023 [17]	Bangladesh	Quantitative (cross-sectional survey)	Moderate risk	Good sample in difficult-to-access refugee camps; limited generalizability beyond Cox’s Bazar.
***** Summan et al., 2022 [7]	India (rural, multi-state)	Quantitative (national survey analysis, decomposition)	Low risk	Robust national survey and facility data; large sample size
***** Paul et al., 2022 [9]	Nepal (Makwanpur District)	Qualitative (IDIs, KIIs, FGDs)	Moderate risk	Rich qualitative depth; district-level scope only.
Novilla et al., 2023 [14]	Multi- countries	Systematic review	Low risk	Comprehensive SR; most evidence, indirect transferability.
***** Al-Haroni et al., 2023 [19]	Malaysia (Ampang district, Kuala Lumpur)	Quantitative (cross-sectional)	Moderate risk	limited generalizability (one district)
***** Chung et al., 2016 [20]	China (Yanbian)	Quantitative (survey + vaccination cards)	Moderate risk	Comparative survey; limited to one prefecture.
***** Zhou et al., 2023 [21]	China (Zhejiang & Henan)	Quantitative (cross-sectional survey)	Low risk	Large survey and generalizability; self-report bias possible.
***** Qayyum et al., 2021 [22]	Pakistan (Rajanpur district)	Qualitative (IDIs, FGDs)	Moderate risk	Qualitative depth; not generalizable quantitatively.
***** Lin et al., 2022 [23]	China (Sichuan, Guangdong, Henan)	Qualitative (interviews)	Moderate risk	Good provider perspective; small sample

Note: Criteria for appraisal were adapted from commonly used frameworks for assessing risk of bias in observational and qualitative studies, including the Newcastle–Ottawa Scale (for cross-sectional studies) and the Critical Appraisal Skills Programme (CASP) checklist (for qualitative research), but applied descriptively to align with the narrative review design. Studies marked with an asterisk (*) were appraised using these criteria. The two systematic reviews (Kalaij et al., 2021 [12]; Novilla et al., 2023 [14]) were appraised for general quality and transparency but were not thematically synthesized. Only primary studies informed the thematic domains.

**Table A3:** Mapping of determinants to thematic domains.

Determinants Identified from Included Studies	Thematic Domains
Maternal education, household wealth, EPI card possession	Socioeconomic and access inequities
Geographic remoteness, transport barriers, rural residence	Socioeconomic and access inequities
Misconceptions about vaccines, fear of side effects, religious objections	Trust, cultural beliefs and social norms
Misinformation, media influence, vaccine controversies (e.g., Dengvaxia® in the Philippines)	Trust, cultural beliefs and social norms
Refugee status (Rohingya in Malaysia, displaced populations in camps)	Migration, refugee, and mobility-related vulnerabilities
Stateless children, left-behind children, seasonal migrants	Migration, refugee, and mobility-related vulnerabilities
Birth order, family size, father’s age, caregiver occupation	Household and caregiver dynamics
Influence of grandmothers, fathers, tribal/community leaders	Household and caregiver dynamics
Facility quality, staff shortages, availability of female vaccinators	Health system and program
Provider communication, follow-up mechanisms, integration with maternal–child health services	Health system and program
